# Matching IoT Devices to the Fog Service Providers: A Mechanism Design Perspective†

**DOI:** 10.3390/s20236761

**Published:** 2020-11-26

**Authors:** Anjan Bandyopadhyay, Vikash Kumar Singh, Sajal Mukhopadhyay, Ujjwal Rai, Fatos Xhafa, Paul Krause

**Affiliations:** 1 Department of Computer Science & Engineering, National Institute of Technology Durgapur, Durgapur 713209, West Bengal, India; ab.15it1102@phd.nitdgp.ac.in (A.B.); sajal@cse.nitdgp.ac.in (S.M.); ur.17u10206@btech.nitdgp.ac.in (U.R.); 2 Amity School of Engineering & Technology Kolkata, Amity University, Kolkata 700157, West Bengal, India; 3 School of Computer Science and Engineering, VIT-AP University, Amaravati 522237, Andhra Pradesh, India; 4 Department of Computer Science, Universitat Politècnica de Catalunya, 08034 Barcelona, Spain; 5 Department of Computer Science, University of Surrey, Guildford GU2 7XH, UK; p.krause@surrey.ac.uk

**Keywords:** Fog computing, matching, mechanism design, IoT devices, truthful, Pareto optimal, truthful mechanism

## Abstract

In the Internet of Things (IoT) + Fog + Cloud architecture, with the unprecedented growth of IoT devices, one of the challenging issues that needs to be tackled is to allocate Fog service providers (FSPs) to IoT devices, especially in a game-theoretic environment. Here, the issue of allocation of FSPs to the IoT devices is sifted with game-theoretic idea so that utility maximizing agents may be benign. In this scenario, we have multiple IoT devices and multiple FSPs, and the IoT devices give preference ordering over the subset of FSPs. Given such a scenario, the goal is to allocate at most one FSP to each of the IoT devices. We propose mechanisms based on the theory of *mechanism design without money* to allocate FSPs to the IoT devices. The proposed mechanisms have been designed in a flexible manner to address the long and short duration access of the FSPs to the IoT devices. For analytical results, we have proved the economic robustness, and probabilistic analyses have been carried out for allocation of IoT devices to the FSPs. In simulation, mechanism efficiency is laid out under different scenarios with an implementation in Python.

## 1. Introduction

With the the ever-increasing growth of Internet of Things (IoT) devices across the globe [1] (CISCO has put forward a figure that shows we could have 50 billions devices ready to be linked by the year 2020 with multiple devices belonging to a person on average [2]); vast amounts of data are to be stacked and handled. This study reveals that, one of the prime factors with IoT devices is of limited processing power and storage. These limitations of the IoT devices give rise to several issues such as reliability, security, performance, and privacy [3]. In order to overcome these issues, one of the potential options for handling the data is to utilize the Cloud platform [4,5]. It provides services to the users without establishing infrastructures to their end.

**Figure 1:**
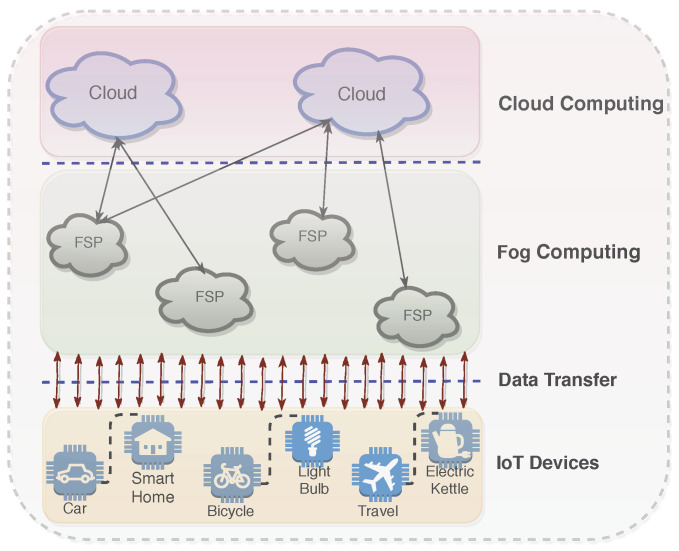
General framework of Internet of Things (IoT) + Fog + Cloud.

The integration of IoT with Cloud brings many advantages to different IoT applications [6]. One of the plausible advantages is managing the huge amount of data generated from sensors and other devices in an efficient and effective manner. These huge amounts of data that are stored in the Cloud are analyzed, and decisions are taken in many real-life scenarios such as telemedicine and patient care. However, sending such a huge amount of data to the Cloud and retrieving them back requires an excessively high network bandwidth. To address this potential point, Fog computing [3,7] could be a sensible approach. It is a newly developed technology by CISCO [8] that will be benefiting several areas, mainly Internet of Things (IoT). Like Cloud computing, Fog computing facilitates services such as data repository and data handling to the IoT users. However, differently from the Cloud, in Fog computing, the processing of data and storage of data is done locally to the available Fog service providers (FSPs). The general framework consisting of Cloud computing, Fog computing, and IoT devices is depicted in Figure 1. Talking in terms of Fog computing, currently, the number of FSPs are not ample. As IoT devices are proliferating, more FSPs may team up to impart services to match the growing demand from IoT devices. The increasing number of FSPs dispenses users (synonymously used with IoT devices) with more preferences, and users will have a chance to speculate about choosing pertinent FSPs. Therefore, for this purpose, the focus should be on developing a good resource allocation mechanism in the context of Fog computing. To address this challenge, in the past, several works have been done that propose resource allocation mechanisms for Fog computing or a similar computing paradigm [9,10,11,12]. However, most of these mechanisms were not designed for the system where the participating agents (such as IoT devices, FSPs, etc.) are strategic in nature. By strategic, we mean that agents will try to game the system in order to gain.

Considering the strategic behavior of the participating agents, previously, research has been carried out that focused on designing truthful (or strategy-proof) mechanisms, where the agents are incentivized for reporting their private information in a truthful manner [5,13,14,15,16,17,18]. The above presented statistics and discussion strongly motivated us to explore the resource allocation problem in the context of Fog computing in strategic setting. A deep learning-based solution coupled with blockchain technology is addressed in [19]. In this paper, one Fog provider has multiple Fog units as the resources, and taking the users as the miners in a blockchain network, the Fog units are leveraged to the miners through a deep learning methodology. Here, the objective is to get the optimal allocation as well as to maximize revenue in terms of payment. Other directions that are playing a pivotal role for Fog computing research include data caching and service offloading [20,21], rendering Quality of Service (QoS) and Quality of Experience (QoE) guarantees mainly in terms of energy efficient design, minimizing delay and congestion in the network, and providing efficient local access for achieving the desired objective in Fog computing [21].

Dissimilar to the above discussion and the literature works discussed (see Section 2), in this paper, the problem of allocating IoT devices in Fog environment is formulated using a game-theoretic idea where money is not involved. In literature, this game-theoretic idea is named a mechanism design without money [22]. More formally, the FSPs provide their services free of cost to the IoT devices. Prior to this, in [23], the resource allocation problem in Fog computing was investigated in a zero budget environment. In this, we have multiple FSPs (processes the data) and multiple IoT devices (collects the data). A single or multiple FSP(s) can handle the data accumulated by the IoT devices, but the immediate question that arises is how the allocation of FSPs should be done to the IoT devices for processing the data. For this purpose, each of the IoT devices provide strict preference ordering (or ranking) over all the available FSPs or the subset of FSPs. Given this setup, the goal is to allocate at most one FSP to each of the IoT devices. Truthful mechanisms were proposed in [23] for allocating FSPs to the IoT devices.

However, one can think for the more general scenario of the problem considered in [23] by relaxing the constraint that each of the available IoT devices (or users) will be ranking all the available FSPs or the subset of available FSPs in strict sense. Some of the IoT devices may be indifferent among the FSPs, so that the preference lists may have ties. Given this setup, the goal is to allocate FSPs to the IoT devices. However, in this paper, the above discussed problem is studied in two different directions: (1) an IoT device is assigned to the FSP for exclusive use for a longer duration (afterwards, it is termed $t_{i}=\infty$), (2) an IoT device is assigned to the FSP for a shorter duration (afterwards, it is termed $t_{i}\neq \infty$). This case where an access of shorter duration is required is more challenging than the case of long duration access. In these contexts, the truthful (or incentive compatible) mechanisms are proposed, motivated by [24,25]. In addition to the truthfulness, all the mechanisms that are sifted here run in polynomial time, which make our proposed mechanisms scalable. Also, the mechanisms support the case of duration without deadlines and duration with the deadlines for allocating IoT devices to the FSPs and thereby making our mechanisms amenable to address the time bar.

It should be noted that the allocation problem considered in this paper arises more each time in many real-life applications and projects, especially with the emergence of 5G technologies, such as from smart cities and smart transportation systems. For instance, the C-Roads Spain project (with participation of IMP-Information Modeling Processing research group InLab of Universitat Polit$\overset{‘}{e}$cnica de Catalunya, Spain), which is part of the European project C-Roads, includes the deployment of intelligent transport systems in five pilot projects throughout the Spanish geography. The aim is to offer warning services to drivers about the potential risks that can be found on the roads such as road works, stationary vehicle, adverse weather conditions, etc. to evaluate the effectiveness of these services. As a second example, a joint EPSRC/Jaguar LandRover funded project with participation of the University of Surrey, UK is developing an innovative architecture for the dynamic migration of workloads across edge/Fog nodes to support a wide range of use cases for connected and autonomous vehicles.

### Our Contributions

**Figure 2:**
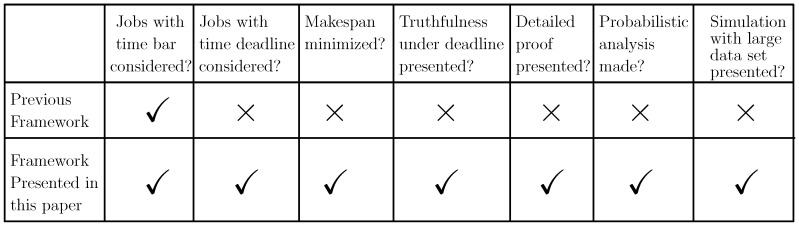
Comparison between the previous framework and the framework presented in this paper.

**Figure 3:**
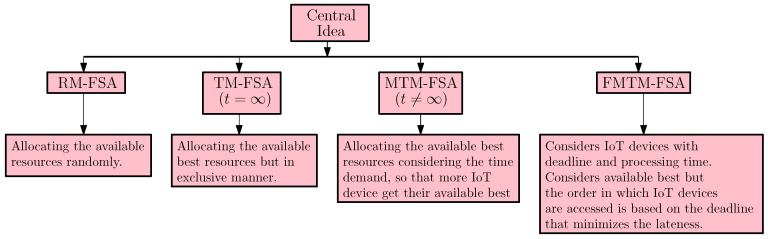
Central idea of the proposed mechanisms.

The main contributions of this paper are the following:

- The proposed mechanisms in this paper are amenable to address the long and short duration access of the FSPs to the IoT devices in a more general setting as the $t_{i}\neq \infty$ situation is extended in a framework that supports the case where the IoT devices posit their demand that encompasses deadline. (The work done in this paper is an extension of the preliminary version of the paper [23] that appeared in The 14th International Conference on P2P, Parallel, Grid, Cloud and Internet Computing (3PGCIC-2019). November 7–9, University of Antwerp, Antwerp, Belgium.) The comparison table comparing the previous framework and the framework presented in this paper is depicted in Figure 2.
  In Figure 2, the comparison between the previous framework and the framework presented in this paper is discussed. In the framework presented in this paper, each of the tasks also have a deadline and the processing time associated with it, whereas in the previous framework, the tasks had only processing time associated with them. In this paper, the makespan resulting after scheduling all the tasks using Further Modified Truthful Mechanism for Fog Service Allocation (FMTM-FSA; the mechanism proposed in the framework presented in this paper) is less as compared to the makespan achieved when scheduled using the Modified Truthful Mechanism for Fog Service Allocation (MTM-FSA; the mechanism proposed in a previous framework).
  In this paper, the truthful mechanism is proposed for the setup consisting of multiple IoT devices and FSPs, where the tasks associated with the IoT devices have both processing time and deadline. Further in this paper, the proof of the lemmas are presented in detailed manner and the extensive probabilistic analysis is carried out. In this paper, the simulation presented in Section 8 is carried out on a large data set, whereas for the previous framework, it is carried out on a small data set. Also, an additional simulation is carried out, where FMTM-FSA and MTM-FSA are compared on the basis of maximum lateness.
- For the case $t_{i}=\infty$, two mechanisms are proposed (one of which is truthful) for allocating FSPs to the IoT devices, namely, Random Mechanism for Fog Service Allocation (RM-FSA) and Truthful Mechanism for Fog Service Allocation (TM-FSA). The truthful mechanism is proposed for the case $t_{i}\neq \infty$, namely, Modified Truthful Mechanism for Fog Service Allocation (MTM-FSA). The central idea of the proposed mechanisms is given in Figure 3.
- The analytical results are provided in terms of the following:
  − Economic robustness (strategy-proofness and Pareto optimality)
  − Probabilistic analysis that envisage the allocation in connection with the preferences furnished by the IoT devices.
- Detailed experimental analysis is carried out based on the following important parameters by considering all cases regarding the availability of FSPs and IoT devices:
  − Efficiency Loss.
  − Best Allocations.

The remaining sections of the paper are described as follows. The prior works are discussed in Section 2. In Section 3, we describe the system model and formulate the problem. The proposed mechanisms for the case $t_{i}=\infty$ are illustrated in Section 4. In Section 5, analysis of the TM-FSA is carried out. The algorithm for the extended version of the problem is depicted in Section 6. The discussed model is further extended in Section 7. The experimental results are discussed in Section 8. Finally, the conclusion and future work are highlighted in Section 9.

## 2. Related Work

As the background of the IoT + Fog + Cloud framework, in the IoT+ Cloud architecture, several works have been done in the direction of modeling the problem of resource allocation through the concept of mechanism design with money (mainly auctions) and mechanism design without money, as the participating agents are strategic in nature. In the scenario where money is involved, in [26], an auction is utilized to allocate the computing capacity of a computer to the users. Based on the demand of the computation time by the users, the payment of the users are decided. In [27], a novel model called Zenith is proposed for allocating computing resources in an edge computing platform that allows service providers to establish resource sharing contracts with edge infrastructure providers apriori.

A similar line of thinking is still relevant in today’s Cloud computing market and will be discussed in many subsequent papers. As the participating agents are rational, they can manipulate their private information to have some extra incentives. In order to tackle such a situation, some truthful mechanisms are discussed [17]. Other potential directions that are significantly important for Fog computing research include data caching and service offloading [20,21], rendering Qos/QoE guarantees for achieving the desired objective in Fog computing as efficiently as possible, incorporating machine learning to efficiently allocate the users to the FSPs and to maximize the revenue of the FSPs. The data caching and service offloading that are discussed in [20,21] mainly discuss how the Fog nodes can be used as a repository to provide a local access to the data or service being accessed repetitively instead of accessing the Cloud infrastructures multiple times for the same and thereby avoiding congestion in the network. The data caching techniques that are rendered in [20,21] can be used in our model to have fair allocation where the assignments that are happening could be stored (as a data cache) and a proportional assignment of the IoT devices to the FSPs could be proffered.

The guarantee regarding QoS/QoE is another facet of Fog computing. By structure, Fog computing is potentially viable to inject QoS/QoE guarantees by providing local accesses to the users rather than communicating every time to the Cloud. In literature, various issues like energy-efficient design, latency, and minimizing congestion as a whole are explored in a reactive (as and when demand comes) or a proactive (in hindsight) manner in general [21,28,29] or considering particular application in mind [30,31].

Moving on to the case where money is not involved in any sense, in the setup of [32], there are multiple users (here, users and IoT devices terms are used interchangeably) and multiple service providers, say *n*, and users provide preference ordering over the available service provider. Here, the goal is to allocate the best available service provider to each of the user. The truthful mechanism is proposed for this discussed setup. Further in [33], the setup discussed in [32] is extended to the case where both the users and the service providers provide strict preference ordering (full or partial preference) over the members of the opposite community. A truthful mechanism is proposed to allocate the services to the users (each user receives a single service).

Coming back to our IoT + Fog + Cloud framework, currently, there are few existing works on the concept of Fog computing [7,34]. For a detailed overview of Fog computing and the research challenges, the readers can go through [3,7,34,35]. In the past, several works have been done that consider the problem of resource allocation in the context of Fog computing but in a nonstrategic setting [9,11]. In [36], in Fog computing, the set of participating agents report the bundle of resources along with the bid values. Given this, the goal is to assign available resources to the participating agents in a conflict-free manner. In [37], in order to schedule the tasks of IoT devices, the idea of data mining is utilized. For this, in order to classify the tasks, a mechanism is proposed named I-apriori (an improved version of the apriori association rules algorithm). In [38], in Fog environment, the problem of scheduling delay-critical IoT devices service is formulated using game theory. For this purpose, an intelligent matching algorithm is proposed that matches the Fog nodes to the IoT devices. The above discussed papers in Fog computing have mainly considered the problems from a monetary perspective.

## 3. System Model

In this section, we present the formal statement of our problem. We consider *n* FSPs and *m* IoT devices. It is considered that m=n. However, for the simulation purpose, we have also considered that m≠n (m>n and m<n). The FSPs are present all the time in order to impart their services on a demand basis. In this model, it is considered that the FSPs and IoT devices are heterogeneous in nature. By heterogeneity, we mean that the FSPs may vary in terms of services provided (some FSPs may provide CPU-related services, some may provide data analytic related facilities, and so on) and IoT devices may vary in terms of type of services requested (CPU related services, data analytic related services, and so on).

Based on the type of services provided by the FSPs and the demand of services by the users, the users and the FSPs are categorized into *k* different categories. Here, the categorization is done on the simple search techniques and given *k* different parameters. *k*-Means clustering or single link clustering using Kruskal’s algorithm or any other clustering (fulfilling some optimization criteria) could also be used depending on the situation and objective we have. The set of *k* different categories is given as $C=\{c_{1},c_{2},\ldots ,c_{k}\}$.

In any category $c_{i}$, the set of FSPs is given as $f_{i}=\left\{f_{i}^{1},f_{i}^{2},\ldots ,f_{i}^{n_{i}}\right\}$; here, $n_{i}$ is the number of FSPs in the *i*th category. In our case, $n_{i}\ll n$ and $\sum_{\forall c_{i}}n_{i}=n$. The set of all the FSPs in *k* different categories is given as $f=\{f_{1},f_{2},\ldots ,f_{k}\}$. In any category $c_{i}$, one of the components that is preserved for any FSP $f_{i}^{j}$ is the amount of time it is available to provide its service and is denoted by $e_{i}^{j}$. For all the FSPs in the *i*th category, it is given as $e_{i}=\left\{e_{i}^{1},e_{i}^{2},\ldots ,e_{i}^{n_{i}}\right\}$. For all the *k* different categories, it is given as $e=\{e_{1},e_{2},\ldots ,e_{k}\}$. On the other hand, in any category $c_{i}$, the set of IoT devices is given as $I_{i}=\left\{I_{i}^{1},I_{i}^{2},\ldots ,I_{i}^{m_{i}}\right\}$; here, $m_{i}$ is the number of IoT devices in the *i*th category such that $m_{i}\ll m$ and $\sum_{\forall c_{i}}m_{i}=m$. Further, each IoT device $I_{i}^{j}$ is characterized by the two parameters: (1) the preference ordering of $I_{i}^{j}$ over the subset of $f_{i}$ represented by the symbol ${≻}_{i}^{j}$ or ${=}_{i}^{j}$ and (2) the time required to complete the desired job of the IoT device $I_{i}^{j}$ (here, job means the desired processing of its collected data) represented by the symbol $t_{i}^{j}$. Now, considering the first parameter, each user has a preference ordering over the subset of member of $f_{i}$. The strict preference ordering of *t*th IoT device $I_{i}^{t}\in I_{i}$ in *i*th category over the set $f_{i}$ is denoted by ${≻}_{i}^{t}$. More formally, $f_{i}^{1}{≻}_{i}^{t}f_{i}^{2}$ means that in *i*th category *t* prefers $f_{i}^{1}$ to $f_{i}^{2}$. The ties in the preference list of *t*th IoT device in $c_{i}$ category over the set $f_{i}$ is denoted by ${=}_{i}^{t}$, where $f_{i}^{1}{=}_{i}^{t}f_{i}^{2}$ means that, in *i*th category, the IoT device $I_{i}^{t}$ prefers equally to $f_{i}^{1}$ and $f_{i}^{2}$. In our setup, $≻=\{{≻}_{1},{≻}_{2},\cdots ,{≻}_{k}\}$ represents a set consisting of the preferences given by the IoT devices in *k* different categories. The symbol ${≻}_{i}$ represents the preference list of the IoT devices in category $c_{i}$ and is given as ${≻}_{i}=\left\{{≻}_{i}^{1},{≻}_{i}^{2},{=}_{i}^{3},\ldots ,{≻}_{i}^{m_{i}}\right\}$. For the second parameter, for any category $c_{i}$, $t_{i}^{j}\in ℜ$. It means that it is just a number, say 10, that depicts the completion time from when it is getting allocated. The unit of the number may be second, minute, hour, etc. depending on the applications.

Given the preference list of the IoT devices, the goal is to allocate at most one FSP to each of the IoT devices. Let us say the resulting allocation vector for all the *k* categories is given as $\alpha =\{\alpha_{1},\alpha_{2},\ldots ,\alpha_{k}\}$, in which $\alpha_{i}$ is the allocation set of the participating agents that belong to the category $c_{i}$ and is denoted as $\alpha_{i}=\left\{\alpha_{i}^{1},\alpha_{i}^{2},\ldots ,\alpha_{i}^{m_{i}}\right\}$.

### 3.1. Required Definitions

In this section, we state the definitions that are relevant to our work in this paper.

**Definition** **1** **(Blocking coalition).** *Instead of participating in the mechanism, if a subset of the IoT devices and the FSPs form a coalition, reassign them and improve their allocation in terms of the preferences they get; then, the coalition formed is thereby termed a blocking coalition.*

**Definition** **2** **(Core allocation).** *Allocations where the agents (IoT devices and the FSPs) cannot improve by reassigning them without participating in the mechanism. In other words, a core allocation ensures an allocation without blocking coalition.*

**Definition** **3** **(Truthful or Incentive compatible).** *A mechanism is truthful if the agents cannot gain by misreporting their true preference ordering.*

**Definition** **4** **(Pareto optimal outcome).** *An outcome (allocation) where we cannot make anyone better off without making someone else worse off.*

## 4. Proposed Mechanisms: RM-FSA and TM-FSA (for $t_{i}=\infty$ Case)

In this section, we present and discuss the two proposed mechanisms, namely, RM-FSA and TM-FSA, for the problem under consideration. The idea behind proposing the RM-FSA for our problem is to better understand the truthful mechanism called TM-FSA motivated by [24,25].

### 4.1. Random Mechanism for Fog Service Allocation (RM-FSA)

In this section, RM-FSA is discussed. First, the underlying idea of RM-FSA is presented. After that, RM-FSA is discussed and presented in a detailed manner.

#### 4.1.1. Outline of RM-FSA

The central idea of RM-FSA is that, for each category $c_{i}$, an IoT device is picked up randomly and an FSP is assigned randomly from the preference list of the IoT device under consideration. Notice that, once the allocation is done, both the IoT device and the FSP are removed from the market. The process iterates until the list of available IoT devices gets exhausted. For any arbitrary category $c_{i}$:

1. In a random order, choose an unassigned IoT device.
2. Go through the preferences of the newly chosen IoT device.
  (a) If the list is not empty, then randomly pick an FSP from the selected IoT device preference list and allocate it. Remove the IoT device along with the allocated FSP from the market.
  (b) Else, remove the unallocated IoT device from market.
3. Until all the IoT devices are not processed, keep repeating the above two steps.

#### 4.1.2. Details of RM-FSA

RM-FSA is a two-phase mechanism: main routine (Algorithm 1) and RM-FSA routine (Algorithm 2). The core allocation property which is defined in Section 3.1 is not guaranteed for RM-FSA. The main reason for constructing the main routine is to perform an allocation process for all the *k* categories. In lines 2–5 of the main routine, for each iteration of the *for* loop of Algorithm 1, the RM-FSA routine is called as depicted in line 3. α maintains the IoT device–FSP pairs for each category in line 4. In line 6, the final allocation α is returned.
 **Algorithm 1:** Main routine (*f*, I, C, ≻)
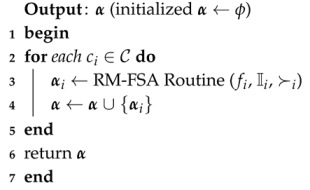


In the RM-FSA routine of Algorithm 2, line 3 is checked for stopping conditions. Lines 4 and 5 choose which IoT device to be processed next. The condition is checked on whether the preference ordering of the selected IoT device is empty in line 6. In line 7, an FSP is randomly picked up from the IoT device *k*’s preference list and is held in $\dot{f}$. The selected IoT device–FSP pairs of the $c_{i}$ category is maintained in $\alpha_{i}$. Using line 9 of the algorithm, the selected IoT device is removed from the market. Similarly, in line 10, the selected FSA is removed from the market. Line 11 removes the selected FSA from the preference ordering of the remaining IoT devices. In line 12, $\dot{f}$ and $\overset{˚}{I}$ are set to ϕ. Next, if the stopping condition in line 6 fails, then lines 14–17 of Algorithm 2 gets executed. Line 15 removes the selected IoT device from the IoT devices list. In line 16, $\overset{˚}{I}$ is set to ϕ. The RM-FSA routine returns the final allocation vector $\alpha_{i}$ in line 19.

### 4.2. Running Time of RM-FSA

The running time of RM-FSA can be deduced by the combination of the running times of main routine and RM-FSA routine. Lines 2–5 in the main routine will execute *k* times. For each iteration of the *for* loop in Algorithm 1, considering RM-FSA routine, line 2 is bounded by the constant time, i.e., O(1). In line 3, the condition is checked $\left(m_{i}+1\right)$ times, as there are $m_{i}$ IoT devices in category $c_{i}$. The *while* loop in lines 3–18 is bounded above by $O(m_{i}^{2}n_{i})$. As in each category $c_{i}$, $n_{i}$ and $m_{i}$ are functions of *n* and *m*, so we have $O(m^{2}n)$.

Hence, the running time of RM-FSA for all the *k* different categories will be $O(km^{2}n)$. If m=n, then the running time of RM-FSA will reduce to $O(kn^{3})$.
**Algorithm 2:** Random Mechanism for Fog Service Allocation (RM-FSA) routine ($f_{i}$, $I_{i}$, ${≻}_{i}$)
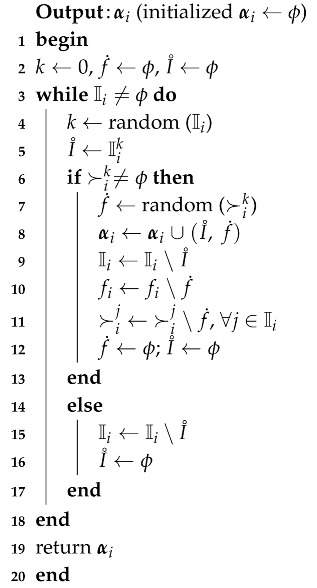


### 4.3. Truthful Mechanism for Fog Service Allocation (TM-FSA)

In this section, first, the underlying idea of the TM-FSA is elaborated. After that, TM-FSA is discussed and presented in detail.

#### 4.3.1. Outline of TM-FSA

The main observation of TM-FSA is that, for each category $c_{i}$, first, the distinct random numbers will be generated between 1 to $m_{i}$ and will be assigned to the IoT devices. Now, based on the random numbers assigned, each time an IoT device will be picked up and will be assigned the best available FSP from its preference list. Once the allocation is done, both the IoT device and the FSA are removed from the market. The process iterates until the IoT devices list gets exhausted. The idea of TM-FSA is depicted below. For any arbitrary category $c_{i}$:

1. IoT devices are numbered with a randomizer.
2. Each of the IoT devices is processed in a non-decreasing order of the number generated by the randomizer.
3. When processed, the match between an FSP and the IoT device is fixed with the most favored FSP available at that time for IoT device.

#### 4.3.2. Detailing of TM-FSA

TM-FSA is a two-phase mechanism: main routine and TM-FSA routine. The main routine performs the allocation process for all the *k* categories. In lines 2–5, for each iteration of the *for* loop of Algorithm 3, the TM-FSA routine is called as depicted in line 3. α maintains the IoT device–FSP pairs that have to be announced finally, and this implementation is done in lines 4 and 6.
**Algorithm 3:** Main routine (*f*, I, C, ≻)
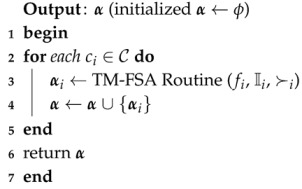


The TM-FSA routine is mainly implemented with the outline presented above with the three steps. IoT devices numbering with a randomizer is provided with lines 3–12. Each of the IoT devices is processed in a non-decreasing order of the number generated by the randomizer and is presented in line 13. Whether the list is exhausted is implemented with line 14. IoT devices are selected sequentially based on the random number assigned in the next line. In line 16, the selected IoT device is stored in $\overset{˚}{I}$. The main part of the third step of the outline is executed in line 17 and line 18, where the match between an FSP and the IoT device is fixed with the most favored FSP available at that time for the IoT device. Line 19 maintains the selected IoT device–FSP pairs in the R data structure. Line 20 removes the selected IoT device and selected FSP from their respective lists. The selected FSP is removed from the preference lists of the remaining IoT devices as depicted in line 21. Line 22 sets $\overset{˚}{I}$ and $\dot{f}$ to ϕ. Next, if the stopping condition in line 17 fails, then lines 24–27 of Algorithm 4 get executed. Line 25 removes the selected IoT device from the IoT devices list. In line 26, $\overset{˚}{I}$ and $\dot{f}$ are set to ϕ. TM-FSA returns the final IoT device–FSP pairs set.

### 4.4. Illustrative Example

**Figure 4:**
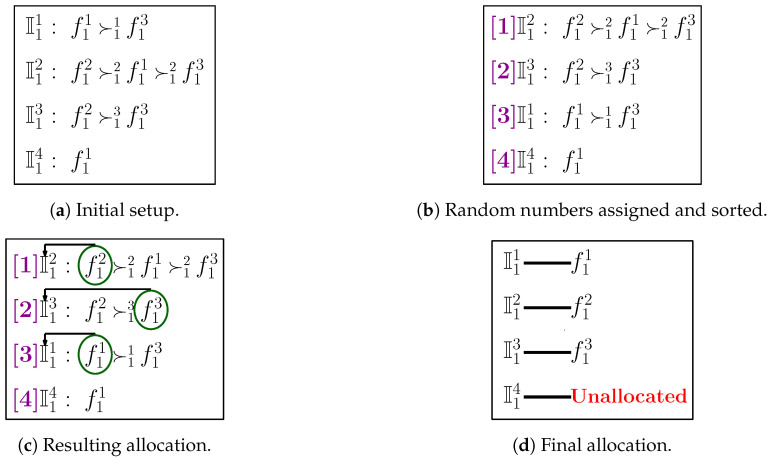
Detailed illustration of TM-FSA.

The detailed functioning of TM-FSA for category $c_{1}$ is depicted in Figure 4. The number of FSPs is 3 (i.e., $n_{1}=3$), and the number of IoT devices is 4 (i.e., $m_{1}=4$). The preference ordering reported by the IoT devices is shown in Figure 4a. Following lines 3–13 of Algorithm 4, the random numbers are generated and assigned to the IoT devices, and based on that, the IoT devices are sorted in ascending order as shown in Figure 4b. From the sorted ordering, first, the IoT device $I_{1}^{2}$ is picked up and assigned the most preferred FSP, i.e., $f_{1}^{2}$. In a similar fashion, the remaining IoT devices $I_{1}^{3}$, $I_{1}^{1}$, and $I_{1}^{4}$ are picked up in the sequence of random number assigned and allocated with the FSPs $f_{1}^{3}$, $f_{1}^{1}$, and none, respectively, as shown in Figure 4c. The final allocation is shown in Figure 4d.

### 4.5. Running Time of TM-FSA

The running time of TM-FSA can be deduced by the combination of the running times of main routine and TM-FSA routine. Lines 2–5 in main routine will execute *k* times. In TM-FSA routine, the random number generator in lines 3–12 of Algorithm 4 is motivated by [39] and it takes $O(m_{i})$ time. In line 13, the IoT devices are sorted in ascending order of the random number assigned and it takes $O(m_{i}\log m_{i})$ time. The allocation carried out in the *while* loop using lines 14–28 is bounded above by $O(m_{i}^{2}n_{i})$. Therefore, the overall running time of the TM-FSA routine for a category $c_{i}$ is given as $O\left(m_{i}\right)+O\left(m_{i}\log m_{i}\right)+O\left(m_{i}^{2}n_{i}\right)=O\left(m_{i}^{2}n_{i}\right)$. As in each category $c_{i}$, $n_{i}$ and $m_{i}$ are functions of *n* and *m*, so we have $O(m^{2}n)$. If m=n, then the running time will be $O(n^{3})$. For *k* different categories, we have the running time of TM-FSA as $O(kn^{3})$.
**Algorithm 4:** Truthful Mechanism for Fog Service Allocation (TM-FSA) routine ($f_{i}$, $I_{i}$, ${≻}_{i}$)
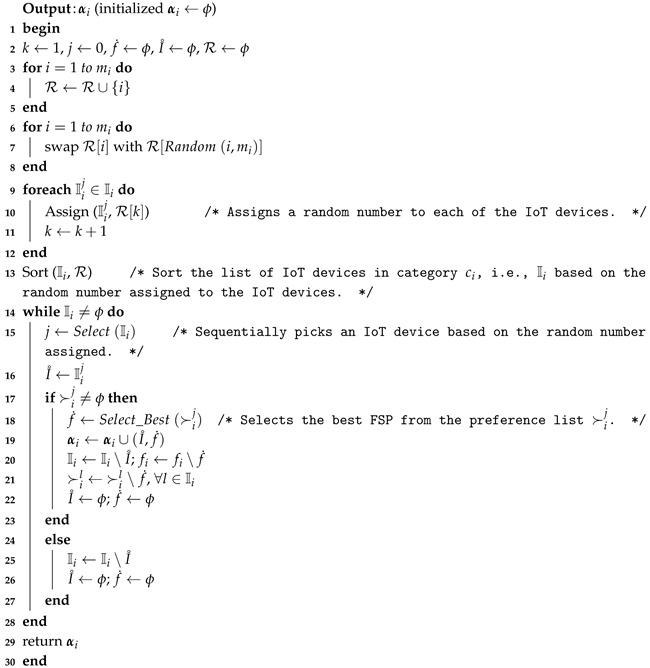


## 5. Analysis of TM-FSA

Now, we will prove some theoretical results about the proposed TM-FSA. It should be noted that, before applying the concept of the draw [25], to which category each IoT device belongs, the duration with which the demand is put forward, and the preferences imparted by it had been sifted and thereby makes our mechanism a two-step process. The two-step process is designed when an exclusive access of the FSP to an IoT device for a longer duration ($t_{i}=\infty$ case) is required and when a flexible access in terms of time is required. For the former case, without changing, the draw can be used to match the FSPs to the IoT devices, but for the later case with flexible time requirement, Draw is applied with little modification. The two-step process of the mechanism entices us to prove that our mechanisms assure truthfulness and Pareto optimality. As we are applying the Draw in our two-fold mechanism, the proof will be similar in nature to that of [25,40].

**Proposition** **1.** *The draw is truthful [25,40].*

**Theorem** **1.** *TM-FSA mechanism is truthful.*

**Proof.**  Fix a category $c_{i}$. The truthfulness of the TM-FSA algorithm depends on the way that every user *i* gets the most ideal decision from the detailed preferences, independent of the classification i∈1,2…k of the user *i*. Notice that the outsider (or the Fog platform) makes a segment for the accessible users and the service providers into various sets in view of their classification. The partitioning of the FSPs set $f=\{f_{1},f_{2},\ldots ,f_{k}\}$ is independent of the partitioning of the accessible users into the set $I=\{I_{1},I_{2},\ldots ,I_{k}\}$. Hence, we demonstrate that, for any user $I_{i}^{\ell }\in I_{i}$, misreporting the private data (for this situation, strict preference over $f_{i}$) will not make the user $I_{i}^{\ell }$ better off.

Each user $I_{i}^{\ell }\in I_{i}$ is assigned a random number and is processed according to that number. It may seem at first that the randomization may adversely affect the truthfulness, but a deeper insight will suggest that it is not. We prove the truthfulness by the induction on *j*. When j=1, the first user (after randomization, we sort the list) in category $c_{i}$ (note that the users belonging to $c_{i}$ are not included in any other category $c_{k}$, and hence, any other category considered will not be affected by this user and vice-versa) will be processed, and according to the algorithm, the best available service provider will be allocated to them. Since no other allocation is done, they will get the first choice. At this point, if they would have given any other rank list, the first preference may come later in rank and would not be the optimal choice. Therefore, the base case holds. Assume it is true up to *j*th users. Now, consider the (j+1)th user. The allocation of the (j+1)th user is independent of the allocation of users that have been allocated before. Therefore, if some preferences of the (j+1)th user have already been allocated to the users processed before, they cannot do anything. Now note that, out of the remaining preferences (if it is not already exhausted), the (j+1)th user gets the best available one by the flow of the algorithm. If, at this point, they alter their preference list, like j=1, the best available choice may be pushed down the line of the rank list and they may not get the best available one, yielding no gain in terms of allocation.

This proves our claim that TM-FSA is truthful. □

**Proposition** **2.** *The draw is Pareto optimal [25,40].*

**Theorem** **2.** *The allocation computed by TM-FSA is Pareto optimal.*

**Proof.**  The proof is by a “greedy stays ahead” kind of argument. Assume that any arbitrary algorithm called ANY is running in parallel with TM-FSA. We will prove that, in any arbitrary iteration, if ANY does not take the policy of TM-FSA, then the user will be worse-off, violating the Pareto optimality condition. Say upto the *i*th iteration, the TM-FSA and ANY output the same allocation. Now, consider the (i+1)th iteration for allocation of the (i+1)th user (note that the (i+1)th user represents a particular category and no user from other category will compete with the (i+1)th user). TM-FSA will allocate, by definition, the best available option to the (i+1)th user. If ANY chooses other policy than this, then the (i+1)th user may not get the best available service provider and definitely will get a lower ranked service provider. Therefore, we can infer that, in any *i*th iteration, TM-FSA will give the best allocation, considering the fact that allocation of the (i+1)th user is independent of allocation of the other users of categories.

Therefore, the allocation given by TM-FSA is Pareto optimal. □

In the context of allocation mechanisms, it would be interesting to estimate the expected number of IoT devices getting their first preference allocated. For TM-FSA, we prove the following result.

**Lemma** **1.** *In TM-FSA, for any category $c_{j}$, the expected number of IoT devices getting their first preference is given by $\frac{m_{j}}{2}$. In other words, $E\left[X_{j}\right]=\frac{m_{j}}{2}$, where $X_{j}$ is the random variable determining the total number of IoT devices getting their first preference.*

**Proof.**  Fix a category $c_{j}$. Let us define the event B as the *i*th agent’s first preference already consumed by any of the (i−1)th agents. In that case, they will not get the best allocation. Further, let us define the event $B_{k}$ as any agent 1≤k<i having the same best preference as the *i*th agent. We can then write

(1) $$Pr\left\{B\right\}=\sum_{k=1}^{i-1}Pr\left\{B_{i}\right\}$$

Equation (1) signifies the fact that whether *i*th agent’s first preference is consumed by the first agent already considered or by the second agent already considered and so on up to the (i−1)th agent, and because of this, we are summing up the probability. One agent’s preference is randomly picked. Therefore, the probability that the *i*th agent’s first preference will become the first preference of any prior agent is $\frac{1}{m_{j}}$, as the *i*th agent’s first preference being the first preference is equally likely. Now, from Equation (1):

(2) $$Pr\left\{B\right\}=\sum_{k=1}^{i-1}\frac{1}{m_{j}}=\frac{i-1}{m_{j}}$$

In Equation (2), we plug the probability that is calculated for the event of any $B_{i}$, and with algebraic manipulation, we get the result. Let us define the event A as the *i*th agent’s first preference surviving.

(3) $$Pr\left\{A\right\}=1-Pr\left\{B\right\}=1-\frac{i-1}{m_{j}}$$

Equation (3) is obtained by the complementary event property. Now, let us define the indicator random variable as $X_{ji}$ = I{ith IoT device of *j*th category being allocated the best preference }

$$I=\left\{\begin{array}{cc} 1, & \mathrm{if}\;\mathrm{ith}\;\mathrm{IoT}\;\mathrm{device}\;\mathrm{is}\;\mathrm{allocated}\\ 0, & \mathrm{otherwise} \end{array}\right.$$

Let us denote the random variable $X_{j}$ as the total number of agents getting their first preference in category $c_{j}$.

$$X_{j}=\sum_{i=1}^{m_{j}}X_{ji}$$

Taking the expectation pf both sides, we get

$$E\left[X_{j}\right]=E\left[\sum_{i=1}^{m_{j}}X_{ji}\right]$$

By linearity of expectation, we get

$$E\left[X_{j}\right]=\sum_{i=1}^{m_{j}}E\left[X_{ji}\right]$$

$$\;=\sum_{i=1}^{m_{j}}Pr\left\{X_{ji}\right\}$$

$$\;=\sum_{i=1}^{m_{j}}Pr\left\{A\right\}$$

$$\;=\sum_{i=1}^{m_{j}}\left(1-\frac{i-1}{m_{j}}\right)$$

$$\;=\sum_{i=1}^{m_{j}}1-\frac{1}{m_{j}}\sum_{i=1}^{m_{j}}\left(i-1\right)$$

$$\;=m_{j}-\frac{1}{m_{j}}\sum_{i=1}^{m_{j}}i+\frac{1}{m_{j}}\sum_{i=1}^{m_{j}}1$$

$$\;=m_{j}-\frac{1}{m_{j}}\cdot \frac{m_{j}\left(m_{j}+1\right)}{2}+\frac{1}{m_{j}}\cdot m_{j}$$

$$\;=m_{j}-\frac{\left(m_{j}+1\right)}{2}+1$$

$$\;=\frac{2m_{j}-m_{j}-1}{2}+1$$

$$\;=\frac{m_{j}-1}{2}+1$$

$$\;=\frac{m_{j}}{2}-\frac{1}{2}+1$$

$$\;=\frac{m_{j}}{2}+\frac{1}{2}$$

$$\;≃\frac{m_{j}}{2}$$

For instance, if $m_{j}=100$, then the value of $E[X_{j}]$ will be approximately 50. It means that approximately half of the IoT devices will be getting their most preferred FSP. □

## 6. Proposed Mechanism: MTM-FSA (for $t_{i}\neq \infty$ Case)

In this section, Modified TM-FSA (MTM-FSA) is discussed. We will first make an observation that will help develop the algorithm in this setting. After that, the underlying idea of the MTM-FSA is elaborated. Next, MTM-FSA is discussed and presented in a detailed manner.

### 6.1. Observation

When a flexible time requirement is put forward by the IoT devices to access the FSPs, then a little care is to be taken for allocating the IoT devices to the FSPs; otherwise, one IoT device will get an entire access to the FSP, excluding others. In the $t_{i}\neq \infty$ case, if we take some care, then more than one IoT devices may be accommodated to the same FSP if they impart the same preference. In this case, we have modified the TM-FSA to accommodate more users to a particular service provider if IoT devices have the same preference. We call this algorithm Modified TM-FSA (MTM-FSA).

### 6.2. Outline of MTM-FSA

In this mechanism, initially, randomizer IoT devices belonging to an arbitrary $c_{i}$ are numbered when they are to be processed. Now, based on the random numbers assigned, each time an IoT device is selected, it is checked whether the preference list of the IoT device is empty. If not, then a check is made on whether the time needed to complete the job of the selected IoT device is less than or equal to the available time of the FSP under consideration. If yes, then the FSP is allocated to the IoT device. Otherwise, the FSP is removed from the list. The process is repeated until the preference list of the IoT device becomes empty, or an allocation is made to the IoT device under consideration. On the other hand, if the preference list of the IoT device is empty, remove the IoT device from the market. Repeat this process until the list of available IoT devices does not get exhausted. Let us have a look at the outline of the proposed mechanism, i.e., MTM-FSA depicted below. For any arbitrary category $c_{i}$:

1. First, $m_{i}$ distinct random numbers are generated and assigned to IoT devices.
2. Next, the IoT devices are sorted in ascending order of the random numbers assigned to them.
3. In each iteration, an IoT device is selected from the sorted ordering and a check is made on whether the preference list of the selected IoT device is empty;
  - If not, then
    **Repeat:**
    (a) Check, whether the time needed to complete the job of the selected IoT device is less than or equal to the available time of the FSP under consideration.
    (b) If yes, then allocate the FSP to the IoT device and reduce the available time of FSP by the amount of time needed to complete the job of the selected IoT device. Remove the IoT device from the market.
    (c) Otherwise, remove the FSP from the preference list of IoT devices under consideration.
    **Until:** The preference list of the IoT device becomes empty, or an allocation is made to the IoT device.
  - Otherwise, remove the IoT device from the market.
4. Repeat step 3 until the list of available IoT devices becomes empty.

### 6.3. Detailing of MTM-FSA

MTM-FSA is a two-phase mechanism: main routine and MTM-FSA routine. The main reason for constructing the main routine is to perform the allocation process for all *k* categories. In lines 1–4, for each iteration of the *for* loop of Algorithm 5, MTM-FSA routine is called as depicted in line 3. α maintains the IoT device–FSP pairs for each category in line 3. In line 5, the final allocation α is returned.
**Algorithm 5:** Main routine (*f*, I, C, ≻, *e*, *t*)
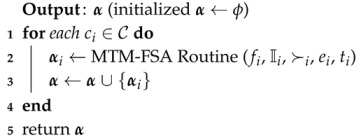


How, with a randomizer, IoT devices are to be numbered and to be accessed one after the other is discussed in lines 3–17 after initializing the required variables in line 2. In lines 18–28, each time, the best available FSP is selected from the preference ordering of $I_{i}^{\ell }$ and the check is made on whether the time needed to complete the job of the selected IoT device is less than or equal to the available time of the FSP under consideration. If yes, then allocate the FSP to the IoT device and reduce the available time of FSP by the amount of time needed to complete the job of the selected IoT device. Otherwise, remove the FSP from the list of IoT devices under consideration. The while loop terminates once the stopping condition in line 18 becomes false. Line 30 removes the considered IoT device from the IoT devices list. Line 31 sets $\overset{˚}{I}$ and $\dot{f}$ to ϕ. The MTM-FSA returns the final IoT device–FSP pairs set $\alpha_{i}$.

### 6.4. Illustrative Example

**Figure 5:**
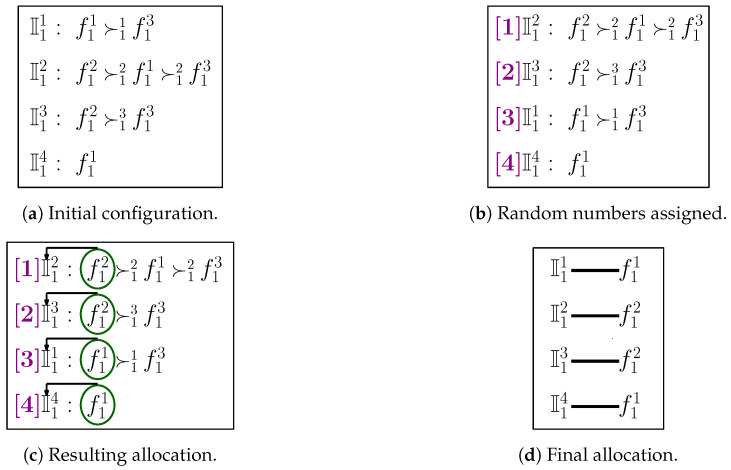
Detailed illustration of MTM-FSA.

The detailed functioning of MTM-FSA for category $c_{1}$ is depicted in Figure 5a. The number of FSPs is assumed to be 3 (i.e, $n_{1}=3)$, and the number of IoT devices is 4 (i.e., $m_{1}=4)$. The preference ordering reported by the IoT devices is shown in Figure 5a. For the running example, it is considered that each FSP will provide its service for 8 h. The times required to complete the jobs of the IoT devices $I_{1}^{1}$, $I_{1}^{2}$, $I_{1}^{3}$, and $I_{1}^{4}$ are 4 h, 5 h, 3 h, and 4 h, respectively. Following lines 3–12 of Algorithm 6, the random numbers are generated and assigned to the IoT devices, and based on that, the IoT devices are sorted in ascending order, as shown in Figure 5b. From the sorted ordering, first, the IoT device $I_{1}^{2}$ is selected and it is seen that FSP $f_{1}^{3}$ is the most preferred FSP, so a check is made on whether that job requested time is 5 ≤ 8 h.
**Algorithm 6:** Modified Truthful Mechanism for Fog Service Allocation (MTM-FSA) routine ($f_{i}$, $I_{i}$, ${≻}_{i}$, $e_{i}$, $t_{i}$)
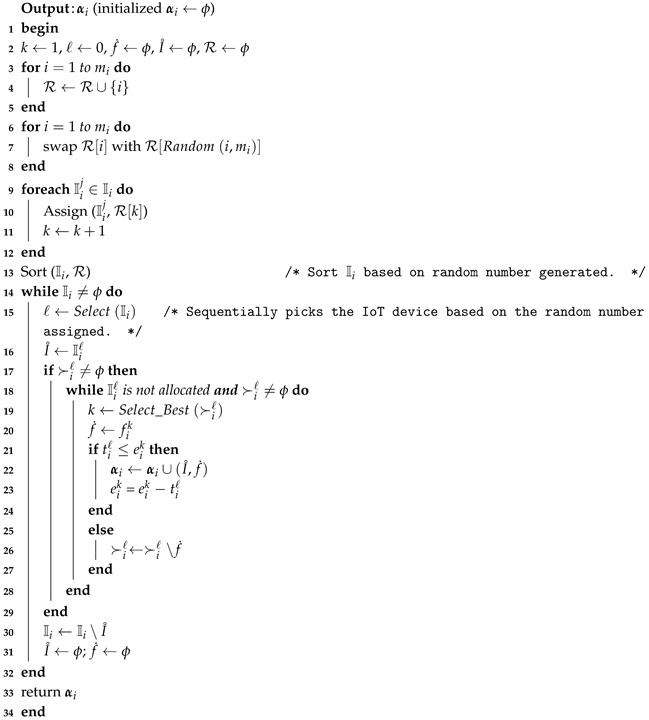


If the answer is *yes*, then the most preferred FSP, i.e., $f_{1}^{2}$, is assigned to $I_{1}^{2}$ for 5 h. Now, the remaining available time of FSP $f_{1}^{2}$ is 3 h. Next, the IoT device $I_{1}^{3}$ will be picked up and it can be seen that the most preferred FSP is $f_{1}^{2}$. Now, the check is made on whether 3≤3 h. If the condition is true, $f_{1}^{2}$ will be assigned to $I_{1}^{3}$. Next, the IoT device $I_{1}^{1}$ is picked up and a test is made on whether 4≤8 h. If the condition is true, $f_{1}^{1}$ will be assigned to $I_{1}^{1}$. Finally, we are left with the IoT device $I_{1}^{4}$. The time required to complete the job of $I_{1}^{4}$ is 4 h. From the preference list of $I_{1}^{4}$, it can be seen that the FSP $f_{1}^{1}$ is the most preferred one. Now, a check is made on whether 4≤4 h. If the condition is true, $f_{1}^{1}$ is allocated to $I_{1}^{4}$. The final allocation is shown in Figure 5d.

### 6.5. Analysis of MTM-FSA

We now prove that MTM-FSA possesses some interesting economic properties such as truthfulness and Pareto optimality, in Theorem 3 and Theorem 4, respectively.

**Theorem** **3.** *MTM-FSA is truthful.*

**Proof.**  Fix a category $c_{i}$, and consider the *i*th user being processed. The question is whether they should provide the preference list truthfully. Our claim is yes. First observe that all the users 1,2,…,i−1 being processed independently of the preference list provided by the *i*th user and the processing is done sequentially. This ensures that no further processing will be made to users 1,2,…,i−1. Some of the choices that are common to $i^{\prime }s$ preference may be taken away by the earlier users processed before the *i*th user and the *i*th user cannot do anything. Now, the proof reduces to whether the *i*th user gets the best available choice when it is their turn in the processing. By construction of the algorithm, $i^{\prime }s$ preference list will be scanned from top to bottom in that order and they will get the next choice when and only when the previous list has been exhausted. Therefore, we can infer that they will always get the best available choice. Therefore, they should report the privately held preference list in a truthful manner. Any possible lie will allocate him a service provider which is not better than the current best. Hence, MTM-FSA is truthful. □

**Theorem** **4.** *MTM-FSA is Pareto optimal.*

**Proof.**  In MTM-FSA, a user is allocated their first choice as long as possible. Otherwise, they are allocated the best available option. At any *i*th stage, if we consider allocating a user by any other algorithm, they have to chose the strategy of the MTM-FSA; otherwise, it will lead to a sub-optimal allocation in terms of choice (the user will be getting a lower ranked service provider than the current best, a worsening effect, and hence violating the Pareto optimal property). Therefore, we conclude that MTM-FSA is Pareto optimal. □

Again, we are interested in estimating the expected number of allocations of first preferences.

**Lemma** **2.** *The following inequality holds:*

(4) $$\sum_{i=0}^{m_{j}-1}i^{k}\leq \frac{{m_{j}}^{k+1}}{k+1}$$

**Proof.**  In the summation $\sum_{i=0}^{m_{j}-1}i^{k}$, $i^{k}$ is a monotonically increasing function, so we can approximate it with the integral as follows:

(5) $$\sum_{i=0}^{m_{j}-1}i^{k}\leq \int_{0}^{m_{j}}x^{k}dx={\left[\frac{x^{k+1}}{k+1}\right]}_{0}^{m_{j}}=\frac{{m_{j}}^{k+1}}{k+1}$$

In Equation (5), the summation is approximated by the integral with the standard inequality $\sum_{k=m}^{n}f\left(k\right)\leq \int_{m}^{n+1}f\left(x\right)\;dx$. □

Next, the result obtained in the above lemma is used for proving the result of Lemma 3.

**Lemma** **3.** *In MTM-FSA, for any category $c_{j}$, the expected number of allocations of the first preference of all the agents will increase based on the number of slots, say k, available to an FSP.*

**Proof.**  Let us first fix slot *k* for a service provider. At the end, we can vary *k* and explore several possibilities of the expected number of allocations of the first preference of all the agents. First, consider the case that the *i*th agent will not get their first preference when being considered for allocation. This will happen when all *k* slots of their preferred service providers are occupied while allocating (i−1) agents prior to that *i*th agent. Let us define event $O_{j}$ as the *j*th slot of the preferred service provider being occupied ∀j and j=1,…,k. When j=1, all (i−1) agents are available and anyone can be allocated to the first slot, i.e., j=1. By Lemma 1, we get $Pr\left\{O_{1}\right\}=\frac{\left(i-1\right)}{m_{j}}$. When j=2, anyone from (i−2) agents may be mapped at the second slot, i.e., j=2. As the first allocation and second allocation are independent, following the standard result, we can write the probability that both the first and second slots are occupied [39]:

(6) $$Pr\left\{O_{1}\cap O_{2}\right\}=Pr\left\{O_{1}\right\}\cdot Pr\left\{O_{2}\right\}$$

By plugging the probabilities of $O_{1}$ and $O_{2}$ in Equation (6), we get

(7) $$\;=\frac{\left(i-1\right)}{m_{j}}\cdot \frac{\left(i-2\right)}{m_{j}}$$

In a similar manner, we can infer the probability that the *k* slots of the preferred service provider of the *i*th agent being occupied is as follows:

(8) $$Pr\left\{O_{1}\cap O_{2}\cap \ldots \cap O_{k}\right\}=\prod_{l=1}^{k}\frac{\left(i-l\right)}{m_{j}}$$

Equation (8) is obtained by extending Equation (6) in a general setting. Now, we define event *M* as the event that all the *k* slots of the preferred service providers are not allocated and, in that case, the *i*th agent will get the first preference.

(9) $$Pr\left\{M\right\}=1-Pr\left\{O_{1}\cap O_{2}\cap \ldots \cap O_{k}\right\}$$

(10) $$\;=1-\prod_{l=1}^{k}\frac{\left(i-l\right)}{m_{j}}$$

Now, define the indicator random variable $X_{i}$ = I{theith agent will get their first preference}

(11) $$I=\left\{\begin{array}{cc} 1, & \mathrm{if}\mathrm{allocated}\\ 0, & \mathrm{otherwise} \end{array}\right.$$

Therefore, $X=\sum_{i=1}^{m_{j}}X_{i}$ counts the number of agents being allocated to their first preference. Taking expectation on both sides, we get

(12) $$\;E\left[X\right]=E\left[\sum_{i=1}^{m_{j}}X_{i}\right]$$

By linearity of expectation, we have

(13) $$\;E\left[X\right]=\sum_{i=1}^{m_{j}}E\left[X_{i}\right]$$

$$\;=\sum_{i=1}^{m_{j}}\left(1-\prod_{l=1}^{k}\frac{i-l}{m_{j}}\right)$$

$$\;=\sum_{i=1}^{m_{j}}1-\sum_{i=1}^{m_{j}}\prod_{l=1}^{k}\frac{i-l}{m_{j}}$$

$$\;=m_{j}-\sum_{i=1}^{m_{j}}\frac{i-1}{m_{j}}\cdot \frac{i-2}{m_{j}}\ldots \frac{i-k}{m_{j}}$$

$$\;=m_{j}-\frac{1}{m_{j}^{k}}\sum_{i=1}^{m_{j}}\left(i-1\right)\cdot \left(i-2\right)\ldots \left(i-k\right)$$

Using Lemma 2, we can write

(14) $$\;E\left[X\right]\geq m_{j}-\frac{1}{{m_{j}}^{k}}\sum_{i=1}^{m_{j}}{\left(i-1\right)}^{k}$$

$$\;=m_{j}-\frac{1}{{m_{j}}^{k}}\sum_{i=0}^{m_{j}-1}i^{k}$$

$$\;=m_{j}-\frac{1}{{m_{j}}^{k}}\cdot \frac{{m_{j}}^{k+1}}{k+1}=m_{j}-\frac{m_{j}}{k+1}$$

If we put k=1, i.e., only one slot, then it is the case of Lemma 1. If k=2, then $E\left[X\right]\geq m_{j}-\frac{m_{j}}{3}=\frac{2m_{j}}{3}$. Therefore, the expected number of allocations of first preference increases. It can even be observed that, with a small *k*, many of the agents will get their first preference, as we will see in next section. □

## 7. Further Extension of Model

In this section, we provide the variant of our proposed model discussed in Section 6. The setup consists of multiple IoT devices and multiple Fog service providers. Each of the IoT devices have a task that is to be done by utilizing the services provided by FSPs. Here, each of the tasks have a deadline along with processing time. The IoT devices provide preference ordering over the FSPs. Given this setup, the goal is to allocate tasks to the service providers in such a manner that minimizes the maximum lateness. In the setup discussed in Section 6, given the preference list of the IoT devices, the order in which the IoT devices will be given a chance for executing their tasks is decided by the random number assigned. The IoT devices are sorted in increasing order of the random number assigned. From the sorted ordering, each time, an IoT device is picked up and the most preferred FSP is assigned for performing the task. However, following the above discussed approach, a problem that may arise is that the IoT devices may not be able to complete their tasks before the deadline of the tasks. Let us understand the problem that may arise with the help of an example. Let us say we have two users with the same first preference for a service provider. The first user needs 2 h to complete the job with a deadline of 3, and second user needs 3 h to complete the job with a deadline of 5. Now, random numbers are generated and are assigned to the IoT devices. Let us say the first user and second user are assigned the random numbers as 2 and 1, respectively. Following the MTM-FSA (discussed in Section 6), first, the second user will be processed and then the first user. In this case, the second user takes 3 h to complete the assigned job, so it will be finished by 3 (starting from 0). It can be seen that a job is completed within the deadline. Next, as the deadline of the first user is 3, starting from 0, it should complete by 3. However, it can be seen that the job of first user started at 3 and will take 2 h. Therefore, it will be finished by 5. Here, both IoT devices completed their jobs, but the first user faces a lateness of 2 h. Now the question is can we reduce the lateness faced by users? The answer to this question is yes, by utilizing the idea of earliest deadline first (see Section 7.1). For the above discussed situation, in this part, we have proposed a truthful mechanism namely Further Modified Truthful Mechanism for Fog Service Allocation (FMTM-FSA).

### 7.1. Proposed Mechanism: FMTM-FSA

Here, the underlying idea of FMTM-FSA is discussed. After that, FMTM-FSA is presented and discussed in a detailed manner.

#### 7.1.1. Outline of FMTM-FSA

In this paper, we have provided the underlying idea of the proposed mechanism. The key idea of FMTM-FSA is that, for each category $c_{i}$, the IoT devices are sorted in increasing order of the deadline of the jobs associated with them. From the sorted ordering, each time, an IoT device will be selected and will be checked on whether the preference list of the IoT device is empty. If not, then the check is made on whether the time needed to complete the job of the selected IoT device is less than or equal to the available time of the FSP under consideration. If yes, then the FSP is allocated to the IoT device. Otherwise, the FSP is removed from the list. The process is repeated until the preference list of the IoT device becomes empty or an allocation is made to the IoT device under consideration. On the other hand, if the preference list of the IoT device is empty, the IoT device is removed from the market. Repeat this process until the list of available IoT devices does not get exhausted. For any category $c_{i}$:

1. First, sort the IoT devices based on the increasing order of the deadline of the jobs associated with them.
2. In each iteration, an IoT device is selected from the sorted ordering.
  - If its preference list is not void, then
    **Repeat:**
    (a) Check whether the time needed to complete the job of the selected IoT device is less than or equal to the available time of the FSP under consideration.
    (b) If yes, then allocate the FSP to the IoT device and reduce the available time of FSP by the amount of time needed to complete the job of the selected IoT device. Remove the IoT device from the market.
    (c) Otherwise, remove the FSP from the preference list of IoT devices under consideration.
    **Until:** The preference list of the IoT device becomes empty, or an allocation is made to the IoT device.
  - Otherwise, remove the IoT device from the market.
3. Repeat step 3 until the list of available IoT devices becomes empty.

#### 7.1.2. Detailing of FMTM-FSA

FMTM-FSA is a two-phase mechanism: main routine and FMTM-FSA routine. The main reason for constructing the main routine is to perform the allocation process for all *k* categories. In lines 1–4, for each iteration of the *for* loop of the Algorithm 7, FMTM-FSA routine is called as depicted in line 3. α maintains the IoT device–FSP pairs for each category in line 3. In line 5, the final allocation α is returned.
**Algorithm 7:** Main routine (*f*, I, C, ≻, *e*, *t*)
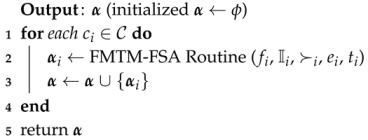


In Algorithm 8, the main trick is applied in line 3 where the IoT devices list $I_{i}$ is sorted based on the deadline of the tasks assigned to them. From line 4, it is clear that the mechanism terminates once the IoT devices list becomes empty. In lines 5–7, selection of the IoT device and availability of its preference list is inquired. In lines 8–18, each time, the best available FSP is selected from the preference ordering of $I_{i}^{\ell }$ and a check is made on whether the time needed to complete the job of the selected IoT device is less than or equal to the available time of the FSP under consideration. If yes, then the FSP is allocated to the IoT device and the available time of FSP is reduced by the amount of time needed to complete the job of the selected IoT device. Otherwise, the FSP is removed from the list of IoT device under consideration. The while loop terminates once the stopping condition in line 8 becomes false. Line 20 removes the considered IoT device from the IoT devices list. Line 21 sets $\overset{˚}{I}$ and $\dot{f}$ to ϕ. FMTM-FSA returns the final IoT device–FSP pairs set $\alpha_{i}$.
**Algorithm 8:** Further Modified Truthful Mechanism for Fog Service Allocation (FMTM-FSA) routine ($f_{i}$, $I_{i}$, ${≻}_{i}$, $e_{i}$, $t_{i}$)
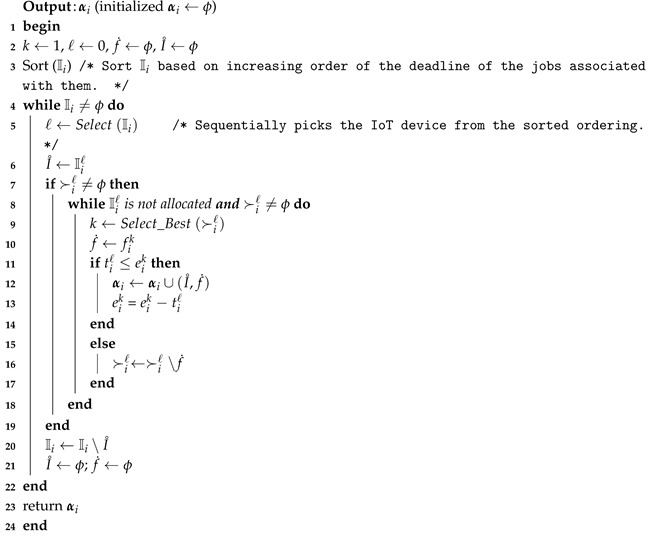


### 7.2. Illustrative Example

**Figure 6:**
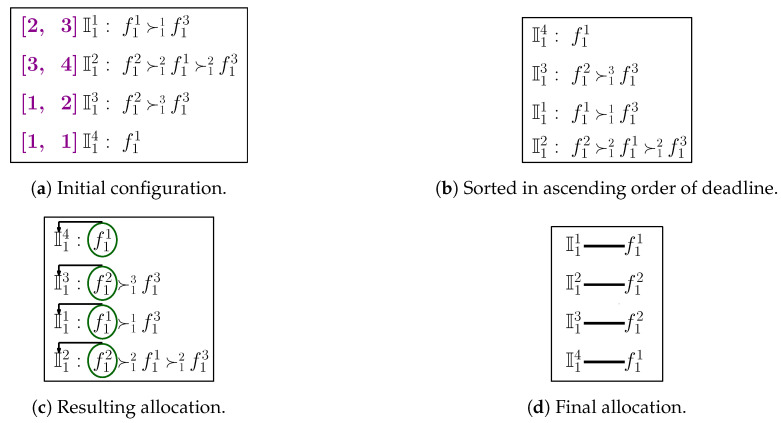
Detailed illustration of FMTM-FSA.

Detailed functioning of FMTM-FSA for category $c_{1}$ is depicted in Figure 6. The number of FSPs is assumed to be 3 (i.e, $n_{1}=3)$, and the number of IoT devices is 4 (i.e., $m_{1}=4)$.

The preference ordering reported by the IoT devices is shown in Figure 6a. For the running example, it is considered that each FSP will provide its service for 8 h. In Figure 6a, the first value represents the time required to complete the jobs of the IoT devices and the second value represents the deadline of the jobs associated with the IoT devices. Following lines 3 of Algorithm 8, the IoT devices are sorted in ascending order of the deadline of the jobs, as shown in Figure 6b. From the sorted ordering, first, the IoT device $I_{1}^{4}$ is selected and it is seen that FSP $f_{1}^{1}$ is the most preferred FSP, so a check is made on whether that job requested time is 1 ≤ 8 h. As the answer is *yes*, then the most preferred FSP, i.e., $f_{1}^{1}$, is assigned to $I_{1}^{4}$ for 5 h. Now, the remaining available time of FSP $f_{1}^{1}$ is 7 h. Next, the IoT device $I_{1}^{3}$ will be picked up, and it can be seen that the most preferred FSP is $f_{1}^{2}$. Now, the check is made on whether 1≤8 h. If the condition is true, $f_{1}^{2}$ will be assigned to $I_{1}^{3}$. Next, the IoT device $I_{1}^{1}$ is picked up and a test is made on whether 2≤7 h. If the condition is true, $f_{1}^{1}$ will be assigned to $I_{1}^{1}$. Finally, we are left with the IoT device $I_{1}^{2}$. The time required to complete the job of $I_{1}^{2}$ is 3 h. From the preference list of $I_{1}^{2}$, it can be seen that the FSP $f_{1}^{2}$ is the most preferred one. Now, a check is made on whether 3≤7 h. If the condition is true, $f_{1}^{2}$ is allocated to $I_{1}^{2}$. The final allocation is shown in Figure 6d.

## 8. Experiments and Results

Here, the experimental results are provided regarding TM-FSA (where full exclusive access is given) and MTM-FSA (for the case $t_{i}\neq \infty$) via simulations (the simulations are done using Python). Considering the first case, TM-FSA is compared with the carefully crafted benchmark mechanism called RM-FSA that is non-truthful in nature. The manipulative behavior of the IoT devices in case of RM-FSA can be seen in the simulation results. On the other hand, the proposed mechanism for the second case, namely MTM-FSA, is compared with the TM-FSA on the ground of number of users getting the most preferred FSP among the available ones. For this case, TM-FSA is considered the benchmark mechanism.

### 8.1. Simulation Setup

**Figure 7:**
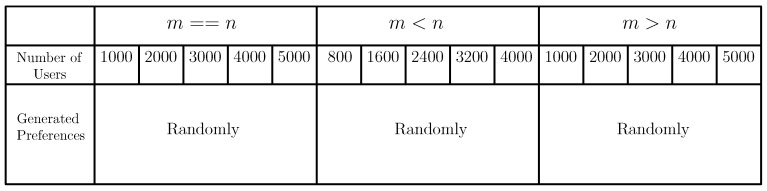
Data set (DS) used for comparing TM-FSA and RM-FSA.

We have done simulations for the 5 different categories of the FSPs and the IoT devices. In each category, a substantial number of FSPs and IoT devices are considered for providing and taking the services. Here, for both cases, the simulations are performed by considering three different scenarios: (1) the total number of IoT devices and FSPs are the same (m=n), (2) the total number of IoT devices is less than the total number of FSPs (m<n), and (3) the total number of IoT devices is greater than the total number of FSPs (m>n). Here, the simulations are accomplished by considering a large data set where the users (IoT devices) could be at most 5000. The preference lists of the users are generated randomly. In the case of extended setup, the processing time and deadline of the tasks are generated using random distribution and normal distribution. Here also, the preference lists of the users are generated randomly. The table shown in Figure 7 depicts the data used for comparing TM-FSA and RM-FSA.

**Figure 8:**
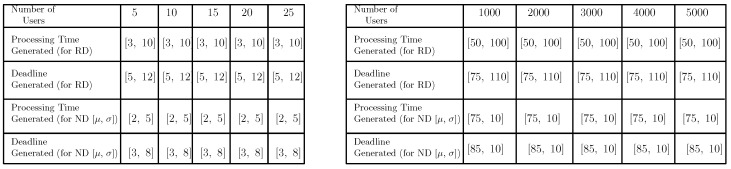
Data set used for comparing FMTM-FSA and MTM-FSA: (**a**) a small DS used for comparing FMTM-FSA and MTM-FSA and (**b**) a large DS used for comparing FMTM-FSA and MTM-FSA.

The table shown in Figure 8 depicts the data used for comparing FMTM-FSA and MTM-FSA.

### 8.2. Performance Evaluation Criteria

Execution of the proposed systems is estimated under the standard of three vital parameters:

- Efficiency Loss (EL): Efficiency loss can be calculated by the difference between the index of the allocated service provider from the user preference list and the index of the most preferred service provider by the user from their preference list.
- Best Allocation (BA): It evaluates the number of the users getting their most preferred service provider from their provided preference list over the accessible number of service providers.
- Lateness: Lateness of the task is defined as the length of time past the deadline of the task. The lateness criteria is considered for the extended setup.

**Figure 9:**
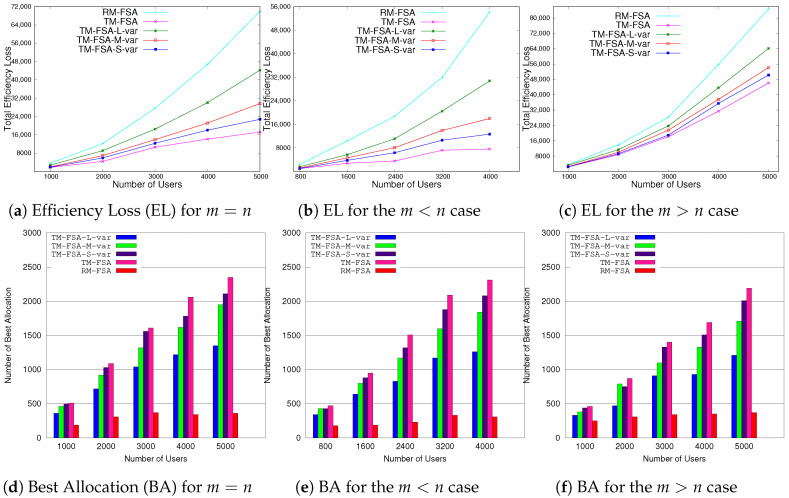
Comparisons of TM-FSA and RM-FSA based on the total efficiency loss and number of best allocation.

**Figure 10:**
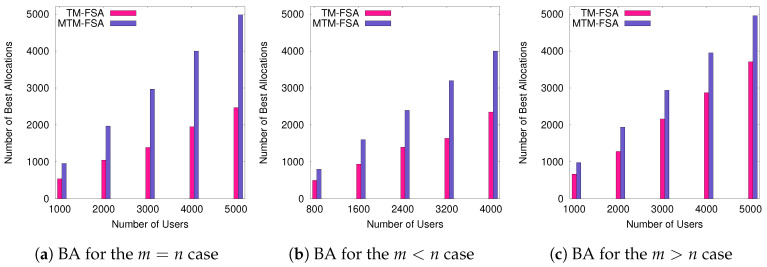
Comparison of TM-FSA and MTM-FSA based on the number of best allocation.

**Figure 11:**
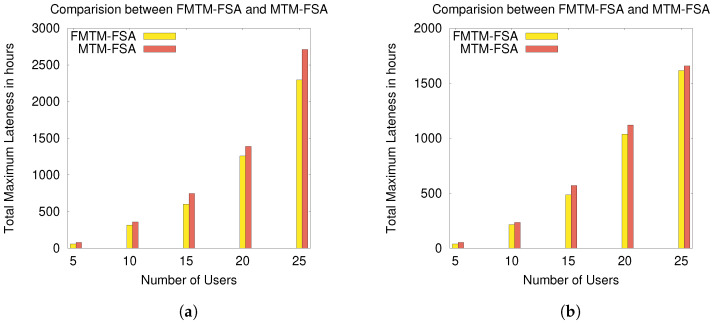
Comparison of FMTM-FSA and MTM-FSA based on lateness for the normal distribution (ND) and random distribution (RD) cases: (**a**) comparing FMTM-FSA and MTM-FSA (ND case) and (**b**) comparing FMTM-FSA and MTM-FSA (RD case).

**Figure 12:**
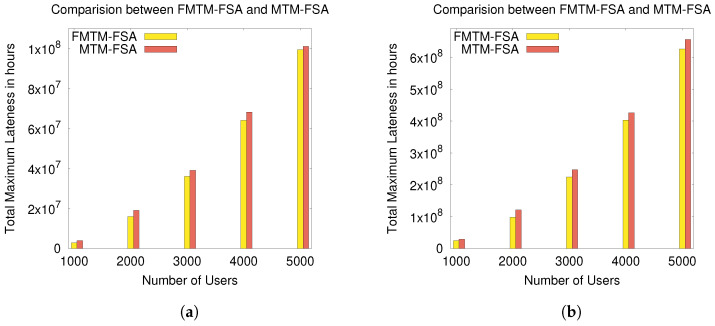
Comparison of FMTM-FSA and MTM-FSA based on lateness for the ND and RD cases: (**a**) comparing FMTM-FSA and MTM-FSA (ND case) and (**b**) comparing FMTM-FSA and MTM-FSA (RD case).

The experimental results according to these performance evaluation criteria are graphically shown in Figure 9, Figure 10, Figure 11 and Figure 12.

### 8.3. Discussion

In order to analyze the manipulative nature of the agents, the simulation is performed in two different directions: (1) when all the agents are reporting their true preference ordering and (2) when part of the total available agents are misreporting their true preference ordering.

Considering the scenario pointed out in point 2 above, for our simulation purpose, we have considered the following:

1. **Small variation (S-var):**$\frac{1}{8}$ of the total available users are manipulating their true preference ordering.
2. **Medium variation (M-var):**$\frac{1}{4}$ of the total available users are manipulating their true preference ordering.
3. **Large variation (L-var):**$\frac{1}{2}$ of the total available users are manipulating their true preference ordering.

### 8.4. Analysis of the Results

In this section, the analyses of the results obtained for two different cases, i.e., $t_{i}=\infty$ and $t_{i}\neq \infty$, are discussed. In the first case, the comparative results of TM-FSA and RM-FSA are depicted in Figure 9a–f. On the other hand, in the second case, the results obtained on comparing MTM-FSA and TM-FSA are given in Figure 10a–c.

- **Case 1:**$t_{i}=\infty$. In Figure 9a–c, it can be seen that the total efficiency loss in case of RM-FSA is more as compared to the total efficiency loss in case of TM-FSA. This is due to the reason that, in contrast to the RM-FSA, TM-FSA allocates the best possible FSP to each of the IoT devices present in the system. In Figure 9a–c, where the IoT devices are not truthfully providing the list that consists of their preferences, then (i) the total efficiency loss of the users in case of TM-FSA-L-var is increased compared to TM-FSA-M-var, (ii) is more than the total efficiency loss of the users in case of TM-FSA-S-var, and (iii) is more that the total efficiency loss of the users in case of TM-FSA. This nature of TM-FSA comes from its construction. Considering the second parameter, i.e., best allocation, in Figure 9d–f, it can be seen that the best allocation of the system in case of RM-FSA is less than the best allocation in the case of TM-FSA. This is due to the reason that TM-FSA allocates the best possible FSP to each of the IoT devices as compared to RM-FSA. In Figure 9d–f, where the list announcement is not done truthfully, we have that the best allocation of the users in the case of TM-FSA-L-var (i) is less than the best allocation of the users in case of TM-FSA-M-var, (ii) is less than the best allocation of the users in the case of TM-FSA-S-var, and (iii) is less than the best allocation of the users in the case of TM-FSA. Again, this is natural from the construction of TM-FSA.
- **Case 2:**$t_{i}\neq \infty$. In Figure 10, MTM-FSA is compared with TM-FSA based on the second parameter, i.e., Best allocation. In Figure 10, it can be seen that the number of best allocations made in the case of MTM-FSA is more as compared to that of TM-FSA in all three different scenarios. This is due to the reason that, in the case of MTM-FSA, no FSP is exclusively given access to a particular user and a single FSP can be allocated to multiple users. If this is the case, then the number of users getting their best preference among the available FSPs gets increased as depicted in Figure 10. Also, these simulation results are supporting the claim made in Lemma 3 that the expected number of allocation of the first preference of all the agents will increase as the available number of slots is increased.

In Figure 11a and Figure 12b, the comparison between MTM-FSA and FMTM-FSA is done on the basis of lateness incurred on scheduling the tasks of the users. The x-axis represents the number of users, and the y-axis represents the total maximum lateness (in hours). In Figure 11a,b, the simulations are performed on a small data set, whereas in Figure 12a,b, the simulations are performed on a large data set. In the case of MTM-FSA, it can be seen that the tasks of the users are scheduled based on the random number assigned to the users. On the other hand, in the case of FMTM-FSA, the tasks of the users are scheduled based on the earliest deadline first. The simulations are done for two different distributions, namely random distribution (RD) and normal distribution (ND). In Figure 11a, for the ND case, the mean (μ) and standard deviation (σ) for the processing time are 5 and 2, respectively. The mean and standard deviation for the deadline are taken as 8 and 3, respectively. In Figure 11b, for the RD case, the processing time and deadline are generated randomly between [3, 10] and [5, 12], respectively. In Figure 11a,b, it can be seen that the total maximum lateness in the case of FMTM-FSA is less as compared to MTM-FSA. This is due to the reason that, in the case of FMTM-FSA, the tasks of the users are scheduled based on the earliest deadline first, whereas in the case of MTM-FSA, the tasks of the users are scheduled based on the random number assigned to the users. Therefore, it can be inferred that FMTM-FSA performs better than MTM-FSA on the basis of maximum lateness. In Figure 12a, for the ND case, the mean and standard deviation for the processing time are 75 and 10, respectively. The mean and standard deviation for the deadline are taken as 85 and 10, respectively. In Figure 12b, for the RD case, the processing time and deadline are generated randomly between [50, 100] and [75, 110], respectively. In Figure 12a,b, it can be seen that the total maximum lateness in the case of FMTM-FSA is less as compared to MTM-FSA due to same reason as above.

## 9. Conclusions and Future Work

In this paper, we have studied the problem of allocating Fog Service Providers (FSPs) to IoT devices in a game-theoretic environment. Here, the FSPs are ranked by the IoT devices in some order and it is not required that all FSPs are ranked. This gives flexibility to the environment. We have considered that the IoT devices provide preference ordering over the subset of available FSPs. We have designed a truthful and Pareto optimal mechanism for allocating at most one FSP to each of the IoT devices. In this case, once the allocation is done, an FSP is exclusively given to a user for a longer period of time. Here, the proposed mechanism, i.e., TM-FSA, is compared with the benchmark mechanism, i.e., RM-FSA, and it is found that TM-FSA performs better than RM-FSA in terms of efficiency loss and number of best allocations. Further, we have extended the setup by considering the case that the participating IoT devices need the FSPs for a specific duration of time from when they are allocated and the demand from the IoT devices for executing the task comprising a deadline along with a processing time. For this setup, we have again designed a truthful and Pareto optimal mechanism. In this case, the proposed mechanism, i.e., FMTM-FSA is compared with MTM-FSA and found that FMTM-FSA performs better than MTM-FSA on the basis of lateness. For all the discussed mechanisms, polynomial time solutions are proposed that signify our mechanisms are scalable.

Till now, we have investigated the case where, given the preference ordering of the IoT devices, each IoT device requests a single FSP. However, in the future, one can consider the case where each IoT device may request for multiple FSPs from its preference list among the available FSPs. Further, in our model, money is not involved. However, by incorporating money in our model, one can prioritize the IoT devices during assignment to the FSPs. A hybrid model can be developed where some assignments may be based without money and some assignments may be based on the priority that could have been imposed to the IoT devices in terms of money.

## Abbreviations

The following abbreviations are used in this manuscript:

IoT	Internet of Things
FSP	Fog Service Provider
RM-FSA	Random Mechanism for Fog Service Allocation
TM-FSA	Truthful Mechanism for Fog Service Allocation
MTM-FSA	Modified Truthful Mechanism for Fog Service Allocation
FMTM-FSA	Further Modified Truthful Mechanism for Fog Service Allocation

## References

[1] Brogi A., Forti S. (2017). QoS-Aware Deployment of IoT Applications Through the Fog. IEEE Internet Things J..

[2] CISCO (2015). Fog Computing and the Internet of Things: Extend the Cloud to Where the Things Are. https://www.cisco.com/c/dam/en_us/solutions/trends/iot/docs/computing-overview.pdf.

[3] Puliafito C., Mingozzi E., Longo F., Puliafito A., Rana O. (2019). Fog Computing for the Internet of Things: A Survey. ACM Trans. Internet Technol..

[4] Bokhari M.U., Makki Q., Tamandani Y.K., Aggarwal V.B., Bhatnagar V., Mishra D.K. (2018). A Survey on Cloud Computing. Big Data Analytics.

[5] Wang Q., Ren K., Meng X. When cloud meets eBay: Towards effective pricing for cloud computing. Proceedings of the IEEE INFOCOM.

[6] Ai Y., Peng M., Zhang K. (2018). Edge computing technologies for Internet of Things: A primer. Digit. Commun. Netw..

[7] Hu P., Dhelim S., Ning H., Qiu T. (2017). Survey on Fog Computing. J. Netw. Comput. Appl..

[8] Bonomi F., Milito R., Zhu J., Addepalli S. (2012). Fog Computing and Its Role in the Internet of Things. Proceedings of the First Edition of the MCC Workshop on Mobile Cloud Computing.

[9] Aazam M., Nam Huh E. Fog Computing Micro Datacenter Based Dynamic Resource Estimation and Pricing Model for IoT. Proceedings of the 2015 IEEE 29th International Conference on Advanced Information Networking and Applications.

[10] Chen W., Paik I., Li Z. (2017). Cost-Aware Streaming Workflow Allocation on Geo-Distributed Data Centers. IEEE Trans. Comput..

[11] Gu Y., Chang Z., Pan M., Song L., Han Z. (2018). Joint Radio and Computational Resource Allocation in IoT Fog Computing. IEEE Trans. Veh. Technol..

[12] Zhang H., Xiao Y., Bu S., Niyato D., Yu F.R., Han Z. (2017). Computing Resource Allocation in Three-Tier IoT Fog Networks: A Joint Optimization Approach Combining Stackelberg Game and Matching. IEEE Internet Things J..

[13] Chawla S., Devanur N.R., Holroyd A.E., Karlin A.R., Martin J.B., Sivan B. (2017). Stability of Service Under Time-of-use Pricing. Proceedings of the 49th Annual ACM SIGACT Symposium on Theory of Computing.

[14] Lucier B., Menache I., Naor J.S., Yaniv J. (2013). Efficient Online Scheduling for Deadline-sensitive Jobs: Extended Abstract. Proceedings of the Twenty-fifth Annual ACM Symposium on Parallelism in Algorithms and Architectures.

[15] Shi W., Wu C., Li Z. (2017). An Online Auction Mechanism for Dynamic Virtual Cluster Provisioning in Geo-Distributed Clouds. IEEE Trans. Parallel Distrib. Syst..

[16] Wang C., Ma W., Qin T., Chen X., Hu X., Liu T.Y. Selling Reserved Instances in Cloud Computing. Proceedings of the 24th International Conference on Artificial Intelligence.

[17] Zhang X., Wu C., Li Z., Lau F.C.M. (2019). A Truthful (1-*ϵ*)-Optimal Mechanism for On-demand Cloud Resource Provisioning. IEEE Trans. Cloud Comput..

[18] Zhu Y., Fu S.D., Liu J., Cui Y. (2018). Truthful Online Auction Toward Maximized Instance Utilization in the Cloud. IEEE/ACM Trans. Netw..

[19] Luong N.C., Jiao Y., Wang P., Niyato D., Kim D.I., Han Z. (2020). A Machine-Learning-Based Auction for Resource Trading in Fog Computing. IEEE Commun. Mag..

[20] Xiang H., Peng M., Cheng Y., Chen H. Joint mode selection and resource allocation for downlink fog radio access networks supported D2D. Proceedings of the 2015 11th International Conference on Heterogeneous Networking for Quality, Reliability, Security and Robustness (QSHINE).

[21] Moura J., Hutchison D. (2019). Game Theory for Multi-Access Edge Computing: Survey, Use Cases, and Future Trends. IEEE Commun. Surv. Tutor..

[22] Nisan N., Roughgarden T., Tardos E., Vazirani V.V. (2007). Algorithmic Game Theory.

[23] Bandyopadhyay A., Xhafa F., Mallik S., Krause P., Mukhopadhyay S., Singh V.K., Maulik U. (2019). A Framework for Allocation of IoT Devices to the Fog Service Providers in Strategic Setting. Advances on P2P, Parallel, Grid, Cloud and Internet Computing (3PGCIC 2019), LNNS.

[24] Gale D., Shapley L.S. (1962). College admissions and the stability of marriage. Am. Math. Mon..

[25] Roughgarden T. (2016). CS269I: Incentives in Computer Science, (Stanford University Course), Lecture #1: The Draw and College Admissions. http://timroughgarden.org/f16/l/l1.pdf.

[26] Sutherland I.E. (1968). A futures market in computer time. Commun. ACM.

[27] Xu J., Palanisamy B., Ludwig H., Wang Q. Zenith: Utility-Aware Resource Allocation for Edge Computing. Proceedings of the 2017 IEEE International Conference on Edge Computing (EDGE).

[28] Shuminoski T., Kitanov S., Janevski T., Molinaro A. (2018). Advanced QoS Provisioning and Mobile Fog Computing for 5G. Wirel. Commun. Mob. Comput..

[29] Bacci G., Belmega E.V., Mertikopoulos P., Sanguinetti L. (2015). Energy-Aware Competitive Power Allocation for Heterogeneous Networks Under QoS Constraints. IEEE Trans. Wirel. Commun..

[30] Tsipis A., Oikonomou K., Komianos V., Stavrakakis I. QoE-Aware Rendering Service Allocation in Fog-Assisted Cloud Gaming Environments. Proceedings of the 2020 5th South-East Europe Design Automation, Computer Engineering, Computer Networks and Social Media Conference (SEEDA-CECNSM).

[31] Madiha H., Lei L., Laghari A.A., Karim S. (2020). Quality of Experience and Quality of Service of Gaming Services in Fog Computing. Proceedings of the 2020 4th International Conference on Management Engineering, Software Engineering and Service Sciences.

[32] Bandyopadhyay A., Mukhopadhyay S., Ganguly U. Allocating resources in cloud computing when users have strict preferences. Proceedings of the 2016 International Conference on Advances in Computing, Communications and Informatics, ICACCI.

[33] Bandyopadhyay A., Mukhopadhyay S., Ganguly U. On free of cost service distribution in cloud computing. Proceedings of the 2017 International Conference on Advances in Computing, Communications and Informatics (ICACCI).

[34] Mahmud R., Kotagiri R., Buyya R., Di Martino B., Li K.C., Yang L.T., Esposito A. (2018). Survey and Future Directions. Internet of Everything: Algorithms, Methodologies, Technologies and Perspectives.

[35] Mouradian C., Naboulsi D., Yangui S., Glitho R.H., Morrow M.J., Polakos P.A. (2018). A Comprehensive Survey on Fog Computing: State-of-the-Art and Research Challenges. IEEE Commun. Surv. Tutor..

[36] Fawcett L., Broadbent M., Race N. Combinatorial Auction-Based Resource Allocation in the Fog. Proceedings of the 2016 Fifth European Workshop on Software-Defined Networks (EWSDN).

[37] Liu L., Qi D., Zhou N., Wu Y. (2018). A Task Scheduling Algorithm Based on Classification Mining in Fog Computing Environment. Wirel. Commun. Mob. Comput..

[38] Arisdakessian S., Wahab O.A., Mourad A., Otrok H., Kara N. (2020). FoGMatch: An Intelligent Multi-Criteria IoT-Fog Scheduling Approach Using Game Theory. IEEE/ACM Trans. Netw..

[39] Cormen T.H., Leiserson C.E., Rivest R.L., Stein C. (2009). Introduction to Algorithms.

[40] Roughgarden T. (2016). CS269I: Incentives in Computer Science (Stanford University Course), Lecture #2: Stable Matching. https://timroughgarden.org/f16/l/l2.pdf.

